# Analysing *Joker*: an attempt to establish diagnosis for a film icon

**DOI:** 10.1192/bjb.2020.146

**Published:** 2021-12

**Authors:** Valentin Yurievich Skryabin

**Affiliations:** Moscow Research and Practical Centre for Narcology, Russia

**Keywords:** Mental health, personality disorders, psychopathology, DSM-5, stigma and discrimination

## Abstract

Todd Phillips's film *Joker*, a 2019 psychological thriller, has stirred up strong reactions to the portrayal of the lead character's mental disorder, which is never specified. I used DSM-5 criteria to study whether Joker/Arthur Fleck showed signs of a real mental disorder. The psychopathology Arthur exhibits is unclear, preventing diagnosis of psychotic disorder or schizophrenia; the unusual combination of symptoms suggests a complex mix of features of certain personality traits, namely psychopathy and narcissism (he meets DSM-5 criteria for narcissistic personality disorder). He also shows the symptoms of pseudobulbar affect due to traumatic brain injury. This apparent co-occurrence of both mental disorder and a neurological condition may be confusing for audiences trying to understand mental illness.

The Joker is considered one of the most recognisable and iconic fictional characters in popular culture and one of the best comic villains.^1^ Since his debut, the Joker has appeared in countless comics, TV shows and films. Like most enduringly popular fictional creations, the Joker has repeatedly morphed his mannerisms, appearance and raison d'être to meet the styles and trends of the time.^1^ He has been adapted to serve as Batman's antagonist in various incarnations, including the 1960s television series *Batman* and 1966 film of the same name, in which he was played by Cesar Romero, and in subsequent films played by Jack Nicholson (*Batman*, 1989), Heath Ledger (*The Dark Knight*, 2008), Jared Leto (*Suicide Squad*, 2016) and Joaquin Phoenix (*Joker*, 2019). Over his 80 years of existence, the Joker's character has gone through numerous significant changes in interpretation, taking a ride between the forces of goofy and evil.

Cesar Romero's slapstick Joker is far from his modern incarnation as an intimidating mass murderer, but Joaquin Phoenix's version has almost the same aesthetic, clearly a deliberate homage to Romero.^2^ Jack Nicholson's Joker shares a similar backstory to Phoenix's, both being normal men with real names (Jack Napier and Arthur Fleck respectively), who, after a formative traumatic incident, come to inhabit the twisted clown persona. Nicholson's Joker was a criminal who was physically and mentally altered after falling into a vat of hazardous chemicals. Heath Ledger's iconic take reinvented the character for a new age, permanently imbuing the clown with angst. Ledger described his character as a ‘psychopathic, mass-murdering, schizophrenic clown with zero empathy’, and Paul Levitz, president of DC Comics in 2002–2009, said, ‘I keep coming back to the way he physically incarnates madness’.^3^ Ledger's incarnation seems to inspire Phoenix's Joker, who is also a social outcast, building on that concept to see the Joker inspire some kind of protest movement.^2^ Jared Leto's take marks the first time that the Joker was almost universally derided, but despite controversial aesthetic choices this incarnation embodies a quintessential component of the Joker's psychology: his ‘super-sanity’ (as stated by Grant Morrison, it refers to the particular state of mind amalgamating elements of Carl Jung’s archetypal trickster figure from mythology, and the corrupting hyper-intellectualism of the dark feminine figures in tragedies (Lady Macbeth, etc.), into Joker’s possession of a higher realm of brain function which we perceive as irrationality; sometimes Joker is even aware that he is a fictional character). Anyway, the differences between different actors and performances should not be overemphasised. In all of his incarnations, the Joker has been a trickster figure who thrives on thwarting expectations and hovering in the uncomfortable, yet mesmerising zone beyond what description can encapsulate.^1^

## Todd Phillips's *Joker*

Todd Phillips's *Joker*, a 2019 psychological thriller film starring Joaquin Phoenix, is a far cry from the previous incarnations, which is stirring up a fierce debate about the portrayal of the character's mental disorder.^4–6^ The director turned a comic icon into a neuropsychological study drama that reflects on how society views and treats the mental health problems of certain individuals. According to some critics, *Joker* runs the risk of being perceived as a glorification of what one angry and disturbed loner is able to accomplish and raises debate over whether the film turns an archetype of a violent man into a heroic figure (intentionally or not).^7^ Andrew A. Nierenberg concluded that ‘Sadly, the movie will increase stigma for those with psychiatric disorders and make people afraid. It will take great efforts to combat this setback in the battle against stigma’.^8^ Furthermore, doctors Annabel Driscoll and Mina Husain wrote in an opinion piece for *The Guardian* soon after the film's release, ‘Severe mental health conditions, such as psychotic illnesses, remain shrouded in stigma and are consistently misrepresented and misunderstood […] Portrayals of mental illness in film can perpetuate unfounded stereotypes and spread misinformation’.^9^ Meanwhile, British neurocriminologist Adrian Raine was impressed by how accurate the film's depiction of the psychology of a murderer was. In an interview with *Vanity Fair*, he described it as ‘a great educational tool’ and stated that he planned to present film clips during his classes.^10^

In general, *Joker* is closer to an art-house film than a typical comic book film, since it shows less explosive action but more social commentary. Unlike other interpretations, we discover a lonely, timid and uncharismatic man. The plot of the film centres on Arthur Fleck, a socially inept party clown and aspiring stand-up comedian living with his ailing mother Penny in Gotham City. Gotham is struggling with crime, unemployment and poverty. Arthur is a middle-aged, underweight and pale-faced man suffering from a medical disorder that causes him to laugh uncontrollably at inappropriate times and he depends on social services for medication. His communication skills are generally poor; he stares at people for too long, uses abnormal facial expressions and misses important interpersonal cues, making others feel discomfort. In the evenings Arthur and his mother watch a TV show with host Murray Franklin, and Fleck imagines himself being on the show and getting everyone's attention. Arthur experiences a romantic feeling towards his neighbour Sophie, but later the film reveals that he was actually imagining all of their dates. During the film, a series of events in close succession ultimately tips him over the edge: Arthur guns down three men on the subway train ride home after he was mocked; his therapy sessions are terminated, he stops taking his medications and later he reveals that Penny adopted him after he was found abandoned. Penny had a mental disorder and her case history records at the mental health clinic mention Arthur having a head injury after she abused him, tied him to a radiator and beat him alongside her abusive boyfriend. Then the dream of the character comes true: he becomes a guest on Murray Franklin's show and asks the host to introduce him as Joker. While it initially seems that Arthur intends to kill himself live on air, he shoots Franklin instead. In the end, we find Joker in a therapy session at the mental health clinic. He is then seen running, dancing and leaving bloodied footprints in the hospital's corridors, implying the psychiatrist's death.

## Diagnosing Joker's mental conditions

Although Arthur's disease remains unspecified throughout the film, we can make some conclusions if we take a look at Joker's symptoms and mental condition throughout the film. During the therapy sessions, Arthur describes his complaints as loneliness, isolation and ‘constant negative thoughts’. An entry in his notebook states that ‘the worst part of having a mental illness is that people expect you to behave as if you don't’. We know that the character attends therapy sessions for a long time and that he is prescribed several psychotropic medications. He even had sufficient insight to say, ‘I need my medication to be increased’. Unfortunately, we have very little information on his family history since he was abandoned as a child. His foster mother has delusional disorder and probably a personality disorder and she underwent in-patient therapy. Arthur was brought up by a foster mother and did not have a father figure. The foster mother herself describes him growing up as a fun and kind boy, but later he was abused and beaten by his adoptive parents and even had a severe head injury. Thus, we can find a number of risk factors for mental disorders in our fictional patient's case history.

### Pseudobulbar affect

In addition, we can assume that Arthur suffers from pseudobulbar affect, or emotional incontinence, which is a disorder of regulation of emotional expression, caused by neurological disease or injury affecting the brain.^11^ Pseudobulbar affect is characterised by sudden, uncontrollable episodes of crying, laughing or both. These episodes are excessive, inconsistent with or disproportionate to circumstances or the patient's underlying mood at the time.^12^ In Joker's case, pseudobulbar affect probably occurred secondary to severe traumatic brain injury (TBI). A number of studies have established that TBI increases the risk of mood disorders, personality changes and substance use disorders.^13–15^ A study by Tateno et al revealed that the prevalence of pathological laughing and crying (PLC) during the first year after TBI was 10.9%, and that compared with patients without PLC, patients with PLC had significantly more depressive, anxious and aggressive behaviours and had poorer social functioning.^16^ This conclusion is consistent with a recently published article that considers neurological aspects of Joker's disease and assumes that he suffers from neuropsychiatric sequelae related to childhood TBI involving the frontotemporal regions and, in particular, the lateral aspect of the left frontal lobe.^17^

### Personality disorders

In general, Arthur appears to have a complex mix of features of certain personality traits, namely narcissism (since he craves attention by any means) and psychopathy (since he demonstrates no empathy for his victims). He also displays some traits of depression, but at the same time, he demonstrates excellent self-control. We can see no evident symptoms of thought disorder; Arthur is a highly motivated lucid thinker, he never tells his therapist about any hallucinations or delusions regarding the neighbour who is the subject of his affections, so their romantic relations and dates may be just his conscious imagination. Such diagnostic vagueness does not allow a diagnosis of psychotic disorder or schizophrenia, although to the viewer's untrained eye Joker may appear a hysterically laughing supervillain who is stereotypically ‘insane’. We also have no evidence to establish a diagnosis of major depressive disorder or bipolar disorder.

#### Narcissistic personality disorder

DSM-5 describes 301.81 Narcissistic Personality Disorder as ‘a pervasive pattern of grandiosity (in fantasy or behavior), need for admiration, and lack of empathy, beginning by early adulthood and present in a variety of contexts’ characterised by at least five of nine criteria.^18^

According to criterion 1, such patients routinely overestimate their abilities and inflate their accomplishments, often appearing boastful and pretentious. They may blithely assume that others attribute the same value to their efforts and may be surprised when the praise they expect and feel they deserve is not forthcoming. Really, Arthur Fleck's life is dominated by his aspiring stand-up career, he craves public attention and likes to imagine himself being on the TV show. Individuals with narcissistic personality disorder are often preoccupied with fantasies of unlimited success, power, brilliance, beauty or ideal love (criterion 2). Throughout the film, Arthur is infatuated with his neighbour, single mother Sophie, and later we recognise that all of their dates were just his fantasies. Individuals with this disorder generally require excessive admiration (criterion 4). Their self-esteem is almost invariably very fragile. They may be preoccupied with how well they are doing and how favourably they are regarded by others. This often takes the form of a need for constant attention and admiration. As we can see throughout the film, Arthur meets this criterion. A sense of entitlement is evident in such individuals' unreasonable expectation of especially favourable treatment (criterion 5). They expect to be catered to and are puzzled or furious when this does not happen. In his fantasies, Arthur charms the audience of the TV show, but when he becomes a guest on the show, he awkwardly tells Murray a joke that he finds funny for its dark humour though nobody else does. After being confronted about this, Arthur grows angrier, resulting in a murder. Finally, our character meets criterion 7, which states that individuals with narcissistic personality disorder generally have a lack of empathy and have difficulty recognising the desires, subjective experiences and feelings of others. Really, Arthur feels no empathy towards his victims. Thus, as we can see, Arthur meets five of the nine criteria, which is enough to establish a diagnosis of 301.81 Narcissistic Personality Disorder.

#### Antisocial personality disorder

At the same time, as I have stated, Arthur has the symptoms of psychopathy. Although psychopathy is not among the ten official personality disorders listed in DSM-5, it is well recognised as a variant of antisocial personality disorder (301.7, according to DSM-5). Indeed, Joker meets a few of the criterion A group of symptoms: he fails to conform to social norms with respect to lawful behaviour, as indicated by repeatedly performing acts that are grounds for arrest; he demonstrates irritability, aggressiveness and disregard for the safety of others, as well as lack of remorse. For this diagnosis to be given, the individual must be at least age 18 years (criterion B) and must have had a history of some symptoms of conduct disorder before age 15 years (criterion C). Arthur is definitely over 18 years of age, but he has no history of symptoms before the age of 15 (or we do not have information about that). For this reason, we cannot establish a diagnosis of 301.7 Antisocial Personality Disorder, according to DSM-5.

## From flawed man to cartoon supervillain

Although the film provides the audience with identifiable components of real mental disorders, in general, the psychopathology that Arthur exhibits is foggy and the combination of his symptoms is unusual. Such diagnostic vagueness helps to create a more relatable character who reflects the burden of any mental disorder; but for the mental health professional it can be confusing and leave the impression that different neurological and mental disorders have been mixed. Actually, the plot moves from a portrait of an individual who is struggling with mental disorder and striving to make a life for himself into pure supervillain caricature. Joker as a character makes it incumbent on the film to drop its pretence at serious character development to enter the comic book mode. Arthur Fleck ceases to be a human being for whom we might feel empathy and descends into a one-dimensional stereotype. The film uses Arthur's childhood trauma as well as his struggle with mental disorder as a means to earn sympathy from the audience, rather than disgust at his actions. It is an age-old cinema psychology cliché: the character hasn't had enough love. Thus, as a character, Arthur appeals deeply to the human tendency towards self-pity. From this point of view, the character's mental illness just happened to be one of the stressors that are the true cause of Arthur becoming the Joker. His mental illness was only important to the overall plot as a way of connecting all the other stressors together. Therefore, to reduce all of Arthur's actions down to his mental health problems is extremely simplistic.

In conclusion, it would be worthwhile to recall an episode from the film. When Arthur's diatribe is booed on the Murray Franklin Show, he tells the audience that ‘humour is subjective’. Likewise, any considered response to divergent interpretations of Arthur Fleck's diagnosis forces specialists to acknowledge their own subjectivity.

## About the author

**Valentin Yurievich Skryabin**, MD, is chair of Department no. 2 at the Moscow Research and Practical Centre for Narcology of the Moscow Department of Public Health, Russia.
